# What is the effect on antibiotic resistant genes of chlorine disinfection in drinking water supply systems? A systematic review protocol

**DOI:** 10.1186/s13750-022-00266-y

**Published:** 2022-03-22

**Authors:** Esfandiar Ghordouei Milan, Amir Hossein Mahvi, Ramin Nabizadeh, Mahmood Alimohammadi

**Affiliations:** 1 Department of Environmental Health Engineering, School of Public Health, Tehran University of Medical Sciences, Tehran, Iran; 2 Center for Solid Waste Research (CSWR), Institute for Environmental Research, Tehran University of Medical Sciences, Tehran, Iran; 3 Center for Air Pollution Research (CAPR), Institute for Environmental Research (IER), Tehran University of Medical Sciences, Tehran, Iran; 4 Center for Water Quality Research, Institute for Environmental Research, Tehran University of Medical Sciences, Tehran, Iran; 5 Health Equity Research Center (HERC), Tehran University of Medical Sciences, Tehran, Iran

**Keywords:** Antibiotic-resistant bacteria (ARB), Antibiotic-resistant genes (ARGs), Drinking water, Water supply, Drinking water treatment plant, Chlorination, Disinfection

## Abstract

**Background:**

Antibiotic-resistant bacteria (ARB) usually enter water sources in different ways, such as via municipal and hospital wastewaters. Because conventional technologies used to treat water inefficient in removing these contaminants (especially antibiotic-resistant genes; ARGs), these contaminants easily enter drinking water distribution networks and pose serious threats to consumers’ health. This study’s main purpose is to systematically investigate the effect of chlorine disinfection on ARGs in drinking water supply systems. This study could play an important role in elucidating the effect of chlorine disinfection on ARGs.

**Methods:**

The systematic review outlining this protocol will be performed according to the Collaboration for Environmental Evidence (CEE) guidelines. The main question is, “what is the effect of chlorine disinfection on ARGs in drinking water supply systems?” For this purpose, the articles will be considered, in which chlorine’s effect on ARGs is investigated. The search includes electronic resources, grey literature, and related websites. Electronic resources include Scopus, PubMed, Embase, Web of Science Core Collection, and Science Direct. After the final search, the obtained articles will be collected in the reference management software (Endnote X8). Upon removing the duplicate articles, the first stage of article screening will be performed based on the title and abstract the articles. In the second stage, the articles obtained from the first screening stage will be screened based on the full text of the articles based on the eligibility criteria. Then, two members of the expert team extract the data. To assess the validity of the articles, bias sources will be determined by an expert team. Biases will be defined according to the criteria designed by Bilotta et al. Finally, a narrative synthesis will be performed for the extracted data; if appropriate data are available, quantitative analysis will also be performed.

**Supplementary Information:**

The online version contains supplementary material available at 10.1186/s13750-022-00266-y.

## Background

Antibiotic-resistant bacteria (ARB) and antibiotic-resistant genes (ARGs) are among the emerging contaminants in water and recently too much attention is paid to these contaminants globally [1, 2]. The widespread use of antibiotics in medicine, livestock breeding, aquaculture and industry have caused the ARB proliferation and release [3, 4]. According to the Centers for Disease Control and Prevention (CDC), in the United States, at least 2 million people are infected with resistant bacteria every year and about 23,000 of these patients lose their lives [5]. According to a review study conducted in 2016, antibiotic resistance caused 700,000 deaths worldwide. Moreover, it is estimated the deaths could reach 10 million by 2050 [6]. In addition, the damage caused by antibiotic resistance to the world economy could reach 100 billion US dollars by 2050 [7–9]. According to World Health Organization (WHO), if immediate action is not taken to combat antibiotic resistance, humans will face deadly infections in the "post-antibiotic era" that can take decades to cure [10]. ARBs enters water sources through municipal wastewater, hospital wastewater, municipal waste, agricultural waste, manure used as fertilizer on agricultural lands, runoff and even the effluent of some industrial treatment plants [11, 12]. After discharge, these contaminants enter surface water and, sometimes, groundwater. Because conventional technologies used for water treatment cannot efficiently remove these contaminants (especially ARGs) [13, 14], and thus, they may easily enter the drinking water distribution networks and turn into a serious threat to consumer health. Some studies have shown that ARBs and ARGs are present in bottled water, wells, rivers, lakes and various sources of drinking water [8]. ARBs and ARGs have also been recorded in drinking water treatment plants and drinking water distribution networks [10, 15]. Genes are transmitted between the bacteria in natural environments and engineering systems such as water and wastewater treatment plants [16]. Water supply systems can act as a suitable reservoir for transferring ARBs and ARGs from the aquatic environment to humans [17]. The main concern is that ARGs (which by themselves pose a little risk) can be easily passed through the water treatment system and transmit resistance to bacteria in the distribution network, including pathogenic bacteria. In this way, they pose serious threats (such as death, deadly and, sometimes, long-term infections) to human health. This expansion of potential bacterial resistance can be accomplished through horizontal transfer of genes (HGT), plasmids, transposons and integrons among different bacterial species [8, 18–21]. In large cities, drinking water is often supplied from surface water resources close to the city after proper and strict treatment processes [22]. The water treatment plant uses various processes such as flocculation, sedimentation, filtration and disinfection to improve water quality. Among these processes, disinfection is the most important process for controlling microorganisms that reach the point of consumption from the treatment plant [23]. Chlorine is used as a disinfectant in drinking water supply systems in many countries due to its cost-effectiveness and simplicity of use [24]. It plays an important role not only in killing bacteria, but also in stabilizing the microbial conditions of water in the distribution network [25, 26]. Recent findings show that chlorine may increase the number of ARGs in drinking water [25, 27, 28]. Depending on the concentration of chlorine in the water, bacteria use different strategies to resist and transmit ARGs [17, 29]. In some studies, the frequency of mobile genetic elements (MGEs), including integrons and plasmids, increases after chlorination, which accelerates ARG transfer [20, 25, 30]. The results of some studies show that chlorination increases ARGs [22, 25, 28], while the results of some other studies indicate a decrease in ARGs after chlorination [31–34]. Therefore, there is debate among researchers and scientists about the effect of disinfectants, especially chlorine, on ARBs and ARGs. Since we did not find any systematic reviews in literature in this field. According to the overview we have done in this area, several review studies have investigated the effect of the chlorination process on ARGs in municipal and hospital wastewater [35–40]. Some reviews have investigated the impact of disinfectants on ARGs in biofilms [41]; others have studied the effect of chlorine on intracellular ARGs [42]; furthermore, others have examined the effectiveness of various treatment technologies and processes in removing ARGs from different aquatic environments [43–46]. Nevertheless, no systematic review article has explicitly investigated the effect of chlorine on ARGs in drinking water supply systems; however, there are several reviews in a similar field that have examined the presence, dissemination and removal of ARG in raw and drinking water. In addition to chlorine, these articles have investigated other processes such as ozone, UV, ultraviolet light and, sometimes, biological activated carbon (BAC) [8, 17, 44, 47, 48]. Because these reviews are not "systematic" and have considered various treatment processes, the effect of chlorine on ARGs has not been studied in details. Thus, the present review can play an important role in elucidating the role of chlorine disinfection on the removal of ARGs. No organization, institution, or individual will be engaged in designing and conducting this study except the authors. Therefore, this study does not have stakeholder engagement.

## Objective of the review

The main purpose of the present work is to investigate systematically the effect of chlorine disinfection (the most widely used disinfection in water supply systems) on ARGs of drinking water supply systems. It is not clear exactly what type of effect chlorine can have on ARGs. Herein, this review explains the effect of chlorine and its compounds on ARGs in drinking water supply systems. It can indicate which ARGs are most present in disinfected (chlorinated) drinking water. It also shows at what dosages chlorine is the most effective in terms of limiting and inactivating ARGs.

### Primary question

“What is the effect of chlorine disinfection in drinking water supply systems on ARGs?” The question includes the following components: Population: Drinking water supply systems such as springs, wells, treatment plants and drinking water distribution networks; Intervention/exposure: Chlorine and chlorine compounds are added to drinking water as a disinfectant; Comparator: The presence of ARGs pre/post chlorination comparison; Outcome: The abundance and presence of ARGs after drinking water chlorination.

### Secondary questions

1. What ARGs are the most commonly present after chlorination?
2. What are the maximum and minimum effects of chlorine dosages on ARGs?
3. What is the optimal chlorine contact time for inactivating or limiting ARGs?

## Methods

This protocol, along with the systematic review outlined, will be performed according to the Collaboration for Environmental Evidence (CEE) guidelines for systematic review and evidence synthesis in environmental management [49], and will be reported according to ROSES reporting standards for systematic review evidence syntheses (see Additional file 1) [50]. This systematic review protocol has been registered in the PROSPERO database (Registration Number: CRD4202124307).

### Searching for articles

In this study, for the scoping exercise, the articles investigating the effects of chlorine on ARGs were considered. At first, a pre-search was performed to determine the approximate number of articles in this field. The articles obtained in this stage were reviewed quantitatively and qualitatively. Also, search terms were identified. After consultation and discussion with the team members, the search strings were revised. High-sensitivity search strategies were designed to identify most of the evidence in this field. The expert team developed a search strategy for each database based on PICO/PECO framework. In the next stage, the search will be based on the published reviews on the topic and suggestions from expert team members in at least four areas, including electronic databases, grey literature, related websites and contact with the authors. The electronic databases included are Scopus, PubMed, Embase, Web of Science Core Collection and Science Direct. We will use Boolean operators to combine search terms and substrings. The 'OR' operator will be used to combine synonym terms that increase search sensitivity. The 'AND' operator will be used to combine the components of a research question (PICO/PECO framework); this will limit the search and increase the search accuracy for retrieving the related articles. It is usually not recommended to use the 'NOT' operator because it may cause the loss of some of the related articles, but to limit a large number of irrelevant articles and specificity of the topic, the 'NOT' operator will be used in this case. Asterisk (*) will be used to include the different search term characters. Finally, if not accessible by the usual retrieval of articles, authors will be directly contacted to request the full texts of their publications [51, 52].

#### Language

The primary search was conducted in Persian and English. However, due to the lack of related studies in Persian, the final search will be limited to English and we will not have a time limit.

#### Estimating the comprehensiveness of the search

To evaluate the performance of the search strategy, a test list (see Additional file 2) of 15 articles was collected from primary search, experts and previous reviews according to the method used by Livoreil et al. [51]. At first, our search strategy could not retrieve the test listing articles; we changed the search strategy again until it retrieved all the test listing articles. Our search strategy was able to find all the test list articles. In addition, we reviewed references to the relevant articles obtained in the search based on the existing search strategy. Most of the relevant articles were found in the references in our search strategy, which confirmed the proper functioning of our search strategy.

#### Publication databases

Search terms will be searched in Scopus based on “TITLE-ABS-KEY”, PubMed based on “MeSH”, Embase based on “EMTREE”, Web of Science Core Collection based on “Topic” and Science Direct based on “Title, abstract or author-specified keywords”. Search strings will be used to search the following five databases (see Additional file 3).

- Scopus (http://www.scopus.com);
- MEDLINE using PubMed (https://pubmed.ncbi.nlm.nih.gov);
- EMBASE (http://www.embase.com);
- Web of Science Core Collection (https://webofknowledge.com); and
- Science Direct (http://www.sciencedirect.com).

Example of a search string in PubMed:

("Drinking Water"[Mesh] OR "Fresh Water"[Mesh] OR "Water"[Mesh]) OR "Water Resources" [Mesh] OR "Water Supply"[Mesh] OR "supply and distribution" [Subheading] OR "Water Purification"[Mesh] OR "Water Quality"[Mesh] OR "Water Wells"[Mesh] OR "Groundwater"[Mesh] OR "Water Pollution"[Mesh]) AND ("Disinfection"[Mesh] OR "Disinfectants"[Mesh] OR "Chlorine"[Mesh] OR "Halogenation"[Mesh]) AND ("Drug Resistance, Multiple, Bacterial"[Mesh] OR "Drug Resistance, Microbial"[Mesh] OR "Drug Resistance, Bacterial"[Mesh] OR "Anti-Bacterial Agents"[Mesh]) NOT "Waste Water"[Mesh] NOT "Sewage"[Mesh].

#### Internet searches

Internet search will be done in Google Scholar. We will search Google Scholar using the Publish or Perish software. The search will be done based on a simplified search string (see Additional file 4) then the results of the first 1000 hits will be downloaded.

#### Specialist searches

Searching for the systematic study will not be limited to electronic databases. Also, it will be done to minimize the bias caused by publishing separate searches in grey literature and websites. In this context, our search will be limited to the keywords “drinking water”, “drinking water treatment plant”, “water supply”, “tap water”, “distribution system”, chlorine*, disinfect*, “antimicrobial resistance”, “antibiotic resistance”, “antibiotic resistant”. These keywords will be used in most searches for grey literature, but are not the same for all the sources and vary according to the source. Searching in grey literature will be done in the following databases and websites [51] (see Additional file 5).

- ProQuest Dissertations and Theses Global;
- Open grey literature in Europe;
- World Alliance against Antibiotic Resistance;
- Center for Antibiotic Resistance Research;
- Centers for Disease Control and Prevention;
- European Committee on Antimicrobial Susceptibility Testing;
- Open Access Theses and Dissertations (OATD);
- Food and Agriculture Organization of the United Nations (FAO);
- British Library for Development Studies (BDLS);
- British Library e-theses online service;
- Directory of Open Access Journals; and
- Bielefeld Academic Search Engine.

#### Supplementary searches

For supplementary searches, citation chasing will be used to identify the potentially relevant studies. If not accessible by the usual retrieval of articles, authors will be directly contacted to request the full texts of their publications.

#### Search updating

Evidence is constantly evolving. New studies are always likely to be undertaken and published. Therefore, if necessary, a search strategy update for all the resources will be performed before the final analysis. The searches will be updated before publication if this systematic review takes more than a year.

## Article screening and study eligibility criteria

### Screening process

**Fig. 1:**
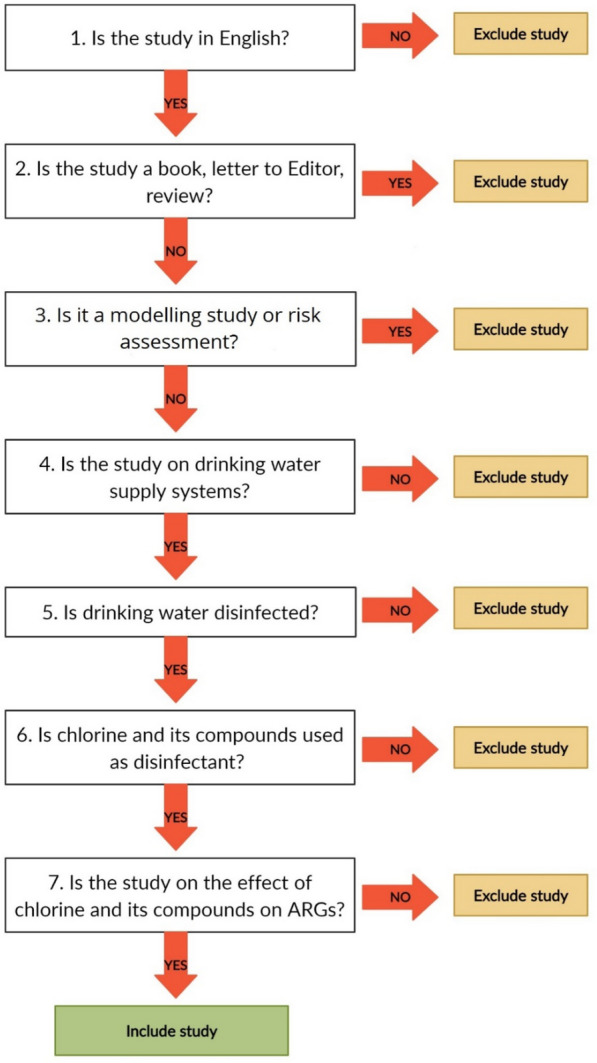
Decision tree for inclusion and exclusion of studies

After the final search, the articles will be collected in the reference management software (Endnote X8). After removing the duplicate articles, screening will be done in two stages (according to Fig. 1). The first screening stage will be based on the title and abstract of the articles. In the second stage and upon full text retrieval, the relevant items will be screened based on the full text criteria. The full text of articles that are not found will be obtained by contacting the authors. For three levels, two team members will perform the screening. In the case of disagreement between the two reviewers, the third reviewer will give the final opinion. All the team members will review articles that have been excluded at the full-text screening level. The project manager will double-check all the excluded articles to verify that no relevant articles are inappropriately excluded [53, 54]. For the procedural independence, we followed the method proposed by Ebrahimi et al. [55]. Systematic reviewers (who have also authored articles to be considered within the review) will not participate in decisions regarding inclusion or study validity assessment of their own work. The reasons for excluding the articles in the screening process (full text) should be reviewed by all the team members and will provide a list of full text articles excluded with reasons for their exclusion.

### Eligibility criteria

Eligibility criteria are described in Table 1. Before screening all the articles (pre-screening), 10% of the articles (minimum 50) randomly will be screened by two reviewers, and Kappa tests will be calculated as a part of consistency checking and to assure consistency among reviewers. The Kappa score of ≤ 0.6 would indicate lack of consistency among reviewers. Eligibility criteria will be clarified and the consistency check will be repeated until the Kappa score is 0.6 or more [52, 56]. According to the eligibility criteria, books will be excluded because they have longer editing and publishing time than most of the books are not available through electronic search and need a manual search, which is very time-consuming and, sometimes, out of reach.

**Table 1:** Eligibility criteria

	Inclusion criteria	Exclusion criteria
Type of study	Original articles, studies presented in theses and conferences	Books, letters to editor, review studies (literature reviews, systematic reviews, and meta-analysis), risk assessment and modeling studies
Language	English	Non-English papers
Population	Water treatment plants, water sources (springs and wells) used as drinking water after disinfection with chlorine and chlorine compounds (such as Cl_2_, Ca(OCl)_2_, NaOCl)	Municipal wastewater, hospital wastewater, sewage, surplus water, runoff
Intervention/exposure	Contact with chlorine and its compounds disinfectant	Contact with other disinfectants (UV, O_3_, etc.)
Outcome	Report of ARGs in chlorinated disinfected water prevalence or concentration of ARGs	No report of ARGs in water before and after chlorine disinfection or only report ARB
Study design	Observational studies (cross-sectional studies) and experimental studies (such as pilot): 1. Study designs with appropriate comparators, including before/after, control/treatment, different interventions (chlorine dosage) as well as studies including both these types of comparisons will be included 2. Studies examining the presence or prevalence of ARGs in chlorinated drinking water	–
Geography	This review is not limited to geographical area (it is global)	–
Period	No time limit	–

## Study validity assessment

The team manager will check again all the articles excluded from the screening process before evaluating the validity of the articles. To evaluate the validity of studies and the internal validity of each included study, sources of bias will be determined by an expert team according to the criteria designed by Bilotta et al. [57, 58] and the criteria defined by Schindler et al. [59]. Table 2 includes the bias assessment framework of the articles. The bias areas include: (1) selection and performance, (2) measurement of outcome, (3) publication and (4) other biases. The score range for each article will be in the range of 0–100. Articles will be classified into three categories with low, medium and high bias. Articles with a score of higher than 67 will be placed in the low bias article class, articles with a score of 33–67 will be placed in the class with moderate bias, and articles with a score below 33 will be placed in the high bias class (see Additional file 6). Articles in category with high bias will be excluded from the quantitative synthesis.

**Table 2:** Bias assessment framework of included articles

Bias area	Characteristic	Bias assessment	Bias score
Selection and performance bias: study design	Sampling	Description of sampling method and transfer of samples to the laboratory	10
		Description of the sampling method or transfer of samples to the laboratory	5
		Method of sampling and transfer of samples to the laboratory is not described	0
	Sample size	Number of samples > 5 or sample size > 10 L	10
		Number of samples < 5 or sample size < 10 L	5
		Lack of sample size and number of samples	0
	Replicates	Replication of samples	Yes [10]
			No [0]
	Study timeframe	Sampling time of more than two seasons (cold and hot seasons, winter and spring or summer)	10
		Sampling time of less than two seasons (hot or cold seasons)	5
		Sampling time is not described	0
Assessment bias: measurement of outcome	Detection of ARGs	The laboratory method and DNA extraction are clear [such as PCR, qPCR, HT-qPCR, high-throughput sequencing (HTS)] Metagenomics	10
		Laboratory methods and DNA extraction are unclear or not described	0
	Chlorine measurement	Using standard methods to measure chlorine (exposure)	10
		Using non-standard or unknown methods to measure chlorine (exposure)	0
	Confounders	Measuring confounding factors and their effects are applied (such as pH, temperature, DO, TOC, phosphate, and nitrate)	10
		Measuring confounding factors, but their effects are not applied	5
		Unmeasured confounders	0
Bias linked to clarity and publication bias	Statistical analyses	Using and describing the statistical data analysis method	Yes [10]
			No [0]
	Reporting bias	All the statistical tests, measurements, and variables mentioned in methods are reported in the results or additional files	Yes [10]
			No [0]
Other biases	Detection bias	Having significant differences in the results before and after chlorination	Yes [10]
			No [0]
	Attrition bias	Measuring the concentration or frequency of ARGs before and after chlorination	Yes [10]
			No [0]
	Research aim consistency	Objectives of the study are clearly stated and the answer is consistent with the objectives	Yes [10]
			No [0]

External validity to determine the generalizability will be assessed using the eligibility criteria in the section above. We will consider for external validity: population (drinking water treatment plant/distribution network/tank/transmission line/tap water), disinfection (chlorine and its compounds), study scale (pilot/field) and presentation of results (absolute versus relative abundance) as well as global susceptibility to bias (low external validity if definitively low internal validity). The critical appraisal could be used to qualify conclusions (i.e., weight studies in the synthesis) if we find a large variance in biases among studies. To assess accuracy in validity assessment, the validity assessment will be conducted on a random sample (at least 20% of the articles) by two reviewers. Disagreements will be resolved after discussion, with the opinion of the third reviewer. Finally, all the included articles will be controlled and confirmed by the team.

## Data coding and extraction strategy

We will extract raw data from appraised full texts. The extracted data will be recorded on a pre-configured Excel spreadsheet (see Additional file 7). In accordance with the pre-designed form author, year of publication, location of study, sample size, type of study (bench, pilot or full scale), laboratory method, type of ARGs, concentration of ARGs, removal rate of ARGs, type of ARBs, type of antibiotic, type and dosage of used disinfectant, type of water such as springs, wells, surface water, groundwater, properties of water such as temperature, pH, DO, total organic carbon (TOC), nitrate and phosphorus as well as existence or absence of biofilm (in the form of existence) will be extracted from the full text of the articles. In the case of incomplete data, the authors of the article will be contacted and the complete data will be obtained. To reduce bias in data reporting and ensure all data are extracted correctly, data extraction will be performed by two team members (at least for 10% of the articles). Moreover, in case of disagreement, the article will be reviewed by the rest of the team [54–56].

## Potential effect modifiers/reasons for heterogeneity

Potential effect modifiers will be identified and recorded from the included articles.

In this study, several factors may cause heterogeneity:

- Type of drinking water source (such as springs, wells, surface water, groundwater, water treatment systems, and drinking water distribution network);
- Properties and characteristics of the drinking water sources (physical, chemical, and biological quality);
- Study design (pilot or field);
- Type of chlorine compounds and dosage of chlorine;
- Monitoring duration;
- Sample size or number of samples;
- Laboratory methods for detection and measuring ARGs [such as PCR, qPCR, HT-qPCR or high-throughput sequencing (HTS)];
- Type of ARGs; and
- Study location.

The list (potential effect modifiers) will be reviewed and completed by the expert team after consultation (if necessary).

## Data synthesis and presentation

Narrative synthesis will be done for all the included articles. The results will be summarized in the tables and figures, and will be accompanied by an interpretation and discussion. Quantitative data analysis will be done for those articles that meet the requirements for quantitative synthesis. ARGs and chlorine doses will be presented based on the mean and standard deviation. Studies with different methods of measuring ARGs concentrations will be analyzed separately. If the studies have sufficient and similar data, meta-analysis (using random-effects models) can be used to analyze the data. Studies with incomplete or missing data will not be included in the meta-analysis. If heterogeneity exists, meta-regression analyses or Cochrane’s Q test will be done depending on the study process to assess between-study heterogeneity. The I^2^ test statistic can be used to measure the extent of this heterogeneity. A funnel plot comparing the study effect size with the standard error may be used to check the publication bias.

## Supplementary Information

**Additional file 1. **ROSES form for the systematic review protocol.

**Additional file 2. **Test list (benchmark articles).

**Additional file 3. **Search strings for electronic database.

**Additional file 4. **Search strings in Google Scholar.

**Additional file 5. **Search strings for grey literature and academic.

**Additional file 6. **Study validity assessment.

**Additional file 7. **Data coding and extraction strategy.

## Data Availability

Not applicable.
