# Supplementary material for: What is the effect on antibiotic resistant genes of chlorine disinfection in drinking water supply systems? A systematic review protocol
Source: Environ Evid. 2022 Mar 22;11:11. doi: 10.1186/s13750-022-00266-y (PMC11378827; doi:10.1186/s13750-022-00266-y)
Supplement: Supplementary file 2 — Additional file 2. Test list (benchmark articles). [file 13750_2022_266_MOESM2_ESM.docx]

README

**Benchmark articles**

This file presents a list of benchmark articles (test list) of known relevance to “**What is the effect on antibiotic resistant genes of chlorine disinfection in drinking water supply systems? A systematic review protocol**”. These should be returned, following a search using the search string, to determine the comprehensiveness of the search.

| NO | Title |
| --- | --- |
| 1 | [Antibiotic resistome alteration by different disinfection strategies in a full-scale drinking water treatment plant deciphered by metagenomic assembly](https://pubs.acs.org/doi/abs/10.1021/acs.est.8b05907)(1) |
| 2 | Chlorination and ultraviolet disinfection of antibiotic-resistant bacteria and antibiotic resistance genes in drinking water(2) |
| 4 | Removal of Antibiotic Resistance Genes and Control of Horizontal Transfer Risk by UV, Chlorination and UV/Chlorination Treatments of Drinking Water(3) |
| 3 | Chlorine injury enhances antibiotic resistance in Pseudomonas aeruginosa through over expression of drug efflux pumps(4) |
| 5 | Comparative removal of antibiotic resistance genes during chlorination, ozonation, and UV treatment(5) |
| 6 | Degradation of extracellular genomic, plasmid DNA and specific antibiotic resistance genes by chlorination(6) |
| 7 | Occurrence and reduction of antibiotic resistance genes in conventional and advanced drinking water treatment processes(7) |
| 8 | Metagenomic assembly provides a deep insight into the antibiotic resistome alteration induced by drinking water chlorination and its correlations with bacterial host changes(8) 2019 .32 |
| 9 | Effect of chlorine on cultivability of Shiga toxin producing Escherichia coli (STEC) and lactamase genes carrying E. coli and Pseudomonas aeruginosa(9) |
| 10 | Long-Term Effects of Residual Chlorine on Pseudomonas aeruginosa in Simulated Drinking Water Fed With Low AOC Medium(10) |
| 11 | Effects of chlorination and ultraviolet light on environmental tetracycline-resistant bacteria and tet (W) in water(11) |
| 12 | High-throughput profiling of antibiotic resistance genes in drinking water treatment plants and distribution systems(12) |
| 13 | Reduction in horizontal transfer of conjugative plasmid by UV irradiation and low-level chlorination(13) |
| 14 | Bacterial Community Shift Drives Antibiotic Resistance Promotion during Drinking Water Chlorination(14) |
| 15 | Metagenomic insights into chlorination effects on microbial antibiotic resistance in drinking water(15) |

**Additional file 2**

The “test list” articles used to evaluate the performance of the search strategy

1. Zhang H, Chang F, Shi P, Ye L, Zhou Q, Pan Y, et al. Antibiotic resistome alteration by different disinfection strategies in a full-scale drinking water treatment plant deciphered by metagenomic assembly. Environmental science & technology. 2019;53(4):2141-50.

2. Destiani R, Templeton M. Chlorination and ultraviolet disinfection of antibiotic-resistant bacteria and antibiotic resistance genes in drinking water. AIMS Environmental Science. 2019;6(3):222-41.

3. Zhang T, Hu Y, Jiang L, Yao S, Lin K, Zhou Y, et al. Removal of antibiotic resistance genes and control of horizontal transfer risk by UV, chlorination and UV/chlorination treatments of drinking water. Chemical Engineering Journal. 2019;358:589-97.

4. Hou A-m, Yang D, Miao J, Shi D-y, Yin J, Yang Z-w, et al. Chlorine injury enhances antibiotic resistance in Pseudomonas aeruginosa through over expression of drug efflux pumps. Water research. 2019;156:366-71.

5. Stange C, Sidhu J, Toze S, Tiehm A. Comparative removal of antibiotic resistance genes during chlorination, ozonation, and UV treatment. International journal of hygiene and environmental health. 2019;222(3):541-8.

6. Zhang M, Chen S, Yu X, Vikesland P, Pruden A. Degradation of extracellular genomic, plasmid DNA and specific antibiotic resistance genes by chlorination. Frontiers of Environmental Science & Engineering. 2019;13(3):1-12.

7. Hu Y, Zhang T, Jiang L, Luo Y, Yao S, Zhang D, et al. Occurrence and reduction of antibiotic resistance genes in conventional and advanced drinking water treatment processes. Science of the Total Environment. 2019;669:777-84.

8. Jia S, Wu J, Ye L, Zhao F, Li T, Zhang X-X. Metagenomic assembly provides a deep insight into the antibiotic resistome alteration induced by drinking water chlorination and its correlations with bacterial host changes. Journal of hazardous materials. 2019;379:120841.

9. Bommer A, Böhler O, Johannsen E, Dobrindt U, Kuczius T. Effect of chlorine on cultivability of Shiga toxin producing Escherichia coli (STEC) and β-lactamase genes carrying E. coli and Pseudomonas aeruginosa. International Journal of Medical Microbiology. 2018;308(8):1105-12.

10. Mao G, Song Y, Bartlam M, Wang Y. Long-term effects of residual chlorine on Pseudomonas aeruginosa in simulated drinking water fed with low AOC medium. Frontiers in microbiology. 2018;9:879.

11. Sullivan BA, Vance CC, Gentry TJ, Karthikeyan R. Effects of chlorination and ultraviolet light on environmental tetracycline-resistant bacteria and tet (W) in water. Journal of environmental chemical engineering. 2017;5(1):777-84.

12. Xu L, Ouyang W, Qian Y, Su C, Su J, Chen H. High-throughput profiling of antibiotic resistance genes in drinking water treatment plants and distribution systems. Environmental Pollution. 2016;213:119-26.

13. Lin W, Li S, Zhang S, Yu X. Reduction in horizontal transfer of conjugative plasmid by UV irradiation and low-level chlorination. Water research. 2016;91:331-8.

14. Jia S, Shi P, Hu Q, Li B, Zhang T, Zhang X-X. Bacterial community shift drives antibiotic resistance promotion during drinking water chlorination. Environmental science & technology. 2015;49(20):12271-9.

15. Shi P, Jia S, Zhang X-X, Zhang T, Cheng S, Li A. Metagenomic insights into chlorination effects on microbial antibiotic resistance in drinking water. Water research. 2013;47(1):111-20.
