# Supplementary material for: What is the effect on antibiotic resistant genes of chlorine disinfection in drinking water supply systems? A systematic review protocol
Source: Environ Evid. 2022 Mar 22;11:11. doi: 10.1186/s13750-022-00266-y (PMC11378827; doi:10.1186/s13750-022-00266-y)
Supplement: Supplementary file 3 — Additional file 3. Search strings for electronic database. [file 13750_2022_266_MOESM3_ESM.docx]

**README**

**Search strings**

This file presents a list of search strings used and formatted for the Scopus, PubMed, Embase, Web of Science Core Collection, and Science Direct databases. The electronic databases included are Scopus, PubMed, Embase, Web of Science Core Collection and Science Direct. We will use Boolean operators to combine search terms and substrings. The 'OR' operator will be used to combine synonym terms that increase search sensitivity. The 'AND' operator will be used to combine the components of a research question (PICO/PECO framework); this will limit the search and increase the search accuracy for retrieving the related articles. It is usually not recommended to use the 'NOT' operator because it may cause the loss of some of the related articles, but to limit a large number of irrelevant articles and specificity of the topic, the 'NOT' operator will be used in this case. Asterisk (*) will be used to include the different search term characters. Search terms will be searched in Scopus based on “TITLE-ABS-KEY”, PubMed based on “MeSH”, Embase based on “EMTREE”, Web of Science Core Collection based on “Topic” and Science Direct based on “Title, abstract or author-specified keywords”.

The Islamic Republic of Iran has subscribed to many journals in databases. We are professors and students of Tehran University of Medical Sciences. This university is affiliated with the Ministry of Health. Every year from January 1 to the end of December. The period when the Ministry of Health subscribes to this database is as follows:

| database | Access details |
| --- | --- |
| Scopus | 2020/1/1-2020/12/30, 2021/1/1-2021/12/30, 2022/1/1-2022/12/30 |
| PubMed | 2020/1/1-2020/12/30, 2021/1/1-2021/12/30, 2022/1/1-2022/12/30 |
| Embase | 2020/1/1-2020/12/30, Access to Embase thanks to a temporary account as a reviewer. |
| Web of Science Core Collection | Inaccessibility, 2021/1/1-2021/12/30, 2022/1/1-2022/12/30 |
| Science direct | 2020/1/1-2020/12/30, 2021/1/1-2021/12/30, 2022/1/1-2022/12/30 |

| Additional file 3  The search strings to execute the searches will be used in five databases: Scopus, PubMed, Embase, Web of Science Core Collection, and Science Direct. | | | | | | |
| --- | --- | --- | --- | --- | --- | --- |
|  | **Population** |  | **Exposure/**  **Intervention** |  | **Outcome** |  |
| Scopus | ( TITLE-ABS-KEY  ( "drinking water" )  OR TITLE-ABS-KEY  ( "drinking water treatment plant" )  OR TITLE-ABS-KEY  ( "DWPT" )  OR TITLE-ABS-KEY  ( "fresh water" )  OR TITLE-ABS-KEY  ( "tap water" )  OR TITLE-ABS KEY  (water )  OR TITLE-ABS-KEY  ( "Water Supply" )  OR TITLE-ABS-KEY  ( "Water Quality" )  OR TITLE-ABS-KEY  ( "Water Well" )  OR TITLE-ABS KEY  ( "Groundwater")  OR TITLE-ABS-KEY  ( "surface water" )  OR TITLE-ABS-KEY  ( "water pollution" )  OR TITLE-ABS-KEY  ( "distribution system" ) | AND | TITLE-ABS-KEY  (disinfect*)  OR TITLE-ABS-KEY  (chlorine*)  OR TITLE-ABS-KEY  (Cl_2_) | AND | TITLE-ABS-KEY  ("antibiotic resistant*")  OR TITLE-ABS-KEY  ("antibiotic resistant* bacteria ")  OR TITLE-ABS-KEY  ("drug resistant*")  OR TITLE-ABS-KEY  ("antimicrobial resistant*")  OR TITLE-ABS-KEY  ("ARG")  OR TITLE-ABS-KEY  ("multidrug resistant*")  OR TITLE-ABS-KEY  ("antibiotic sensitive*") | AND NOT TITLE-ABS-KEY  ( "waste water" )  AND NOT TITLE-ABS-KEY  ( "sewage water" )  AND NOT TITLE-ABS-KEY  ( "wastewater treatment plant" )  AND NOT TITLE-ABS-KEY  ( "municipal wastewater" )  AND NOT TITLE-ABS-KEY  ( effluent )  AND NOT TITLE-ABS-KEY  ( sludge )  AND NOT TITLE-ABS-KEY  ( "liquid waste" ) ) |
| PubMed | ("Drinking Water"[Mesh]  OR  "Fresh Water"[Mesh]  OR  "Water"[Mesh])  OR  "Water Resources" [Mesh]  OR  "Water Supply"[Mesh]  OR  "supply and distribution" [Subheading] OR  "Water Purification"[Mesh]  OR  "Water Quality"[Mesh]  OR  "Water Wells"[Mesh]  OR  "Groundwater"[Mesh]  OR  "Water Pollution"[Mesh]) | AND | ("Disinfection"[Mesh]  OR  "Disinfectants"[Mesh]  OR  "Chlorine"[Mesh]  OR  "Halogenation"[Mesh]) | AND | ("Drug Resistance, Multiple, Bacterial"[Mesh]  OR  "Drug Resistance, Microbial"[Mesh]  OR  "Drug Resistance, Bacterial"[Mesh]  OR  "Anti-Bacterial Agents"[Mesh]) | NOT  "Waste Water"[Mesh]  NOT  "Sewage"[Mesh]) |
| Embase | 'Drinking water'/exp  OR 'fresh water'/exp  OR 'tap water'/exp  OR 'water treatment'/exp  OR 'water supply'/exp  OR 'supply and distribution'/exp  OR'water transport'/exp  OR 'water quality'/exp  OR 'well water'/exp  OR 'ground water'/exp  OR 'surface water'/exp  OR 'water pollution'/exp | AND | 'disinfection'/exp  OR  'disinfectant agent'/exp  OR  'chlorine'/exp  OR  'chlorination'/exp | AND | 'drug resistance'/exp  OR  'antibiotic resistance'/exp  OR  'multidrug resistance'/exp  OR  'antibiotic sensitivity'/exp | NOT  'waste water'/exp  NOT  'municipal wastewater'/exp  NOT  'liquid waste'/exp  NOT  'sewage'/exp  NOT  'effluent'/exp  NOT  'sludge'/exp |
| Web of Science Core Collection | TS=("drinking water" OR "drinking water treatment plant" OR "DWTP" OR "freshwater" OR "tap water" OR water OR "Water Supply" OR "Water Quality" OR "Water well" OR "Groundwater" OR "Surface water" OR "water pollution" OR "distribution system") | AND | TS= (disinfect* OR chlorin* OR cl2 ) | AND | TS= ("antibiotic resistan*" OR "antibiotic resistan* bacteria" OR "drug resistan*" OR "antimicrobial resistan*" OR "ARG" OR "multidrug resistan*" OR "resistan* gene" OR “antibiotic sensitive”) | NOT  TS=("wastewater" OR "waste water" OR "sewage" OR "wastewater treatment plant" OR "municipal wastewater" OR effluent OR sludge OR "liquid waste") |
| Science Direct | 1: Title, abstract or author-specified keywords:("drinking water" OR "tap water" OR "distribution system")  2: Title, abstract or author-specified keywords:("drinking water treatment plant" OR freshwater OR "water quality" OR "drinking water") | AND  AND | Title, abstract or author-specified keywords: (chlorine OR chlorination OR disinfectant)  Title, abstract or author-specified keywords: (chlorination OR disinfection) | AND  AND | Title, abstract or author-specified keywords: ("antibiotic resistant" OR "antibiotic resistance" OR "resistance genes")  Title, abstract or author-specified keywords:("antibiotic resistant" OR "antimicrobial resistant" OR "antibiotic resistance" ) | - |
