# Supplementary material for: What is the effect on antibiotic resistant genes of chlorine disinfection in drinking water supply systems? A systematic review protocol
Source: Environ Evid. 2022 Mar 22;11:11. doi: 10.1186/s13750-022-00266-y (PMC11378827; doi:10.1186/s13750-022-00266-y)
Supplement: Supplementary file 4 — Additional file 4. Search strings in Google Scholar. [file 13750_2022_266_MOESM4_ESM.docx]

**README**

Internet search will be done in Google Scholar. We will search Google Scholar using the Publish or Perish software. Each of the following search strings in the mentioned software will be searched based on the “title”. Finally results of the search strings will be collected in the reference management software (Endnote X8), and then duplicates will be removed.

| **Internet search** | **Search strings in the title** |
| --- | --- |
| Google Scholar | water and disinfection and “antibiotic resistance”  water and disinfection and “antibiotic resistant”  water and disinfectant and “antibiotic resistance”  water and chlorine and “antibiotic resistance”  water and chlorine and “antibiotic resistant”  water and chlorination and “antibiotic resistance”  water and chlorination and “antibiotic resistant”  chlorination and “antibiotic resistance”  disinfectant and “antibiotic resistance”  disinfection and “antibiotic resistance”  “drinking water” and “antibiotic resistance” |
