# Supplementary material for: What is the effect on antibiotic resistant genes of chlorine disinfection in drinking water supply systems? A systematic review protocol
Source: Environ Evid. 2022 Mar 22;11:11. doi: 10.1186/s13750-022-00266-y (PMC11378827; doi:10.1186/s13750-022-00266-y)
Supplement: Supplementary file 5 — Additional file 5. Search strings for grey literature and academic. [file 13750_2022_266_MOESM5_ESM.docx]

**README**

**Search strings**

In this context, our search will be limited to the keywords “drinking water”, “drinking water treatment plant”, “water supply”, “tap water”, “distribution system”, chlorine*, disinfect*, “antimicrobial resistance”, “antibiotic resistance”, “antibiotic resistant”. These keywords will be used in most searches for grey literature, but are not the same for all the sources and vary according to the source. Searching in grey literature will be done in the following databases and websites.

Searching in grey literature will be done in the following databases and websites (not comprehensive)

| literature sources | Search strings |
| --- | --- |
| ProQuest Dissertations and Theses Global (via ProQuest, 1637–present) or PQDT Open | ("Drinking Water" OR "drinking water treatment plant" OR "Water Supply" OR "tap water") AND (chlorination OR disinfection) AND ("antimicrobial resistance" OR "antibiotic resistance" OR “antibiotic resistance gens”) NOT (“sewage” OR “sludge” OR “clinical” OR “waste water” OR “wastewater”)  <https://www.proquest.com> |
| Open grey literature in Europe  (Grey net) | (“drinking water” OR “drinking water treatment plant” OR “water supply”) AND (chlorine* OR disinfect*) AND (“antimicrobial resistance” OR “antibiotic resistance” OR “antibiotic resistant”) NOT “wastewater” NOT sewage  <http://www.opengrey.eu> , <https://easy.dans.knaw.nl> |
| World Alliance against Antibiotic Resistance  <http://resistancecontrol.info/>  <http://resistancecontrol.info>,  <https://waaar.org/en>, | 1. antibiotic resistance gens in drinking water  <http://resistancecontrol.info/2019-contents-list> , <http://resistancecontrol.info/amr-control-2018-contents>, <http://resistancecontrol.info/2018-contents-french-edition>, <http://resistancecontrol.info/2017-contents-list>, <http://resistancecontrol.info/2016-toc>, <http://resistancecontrol.info/2016-toc>, etc. |
| Centre for Antibiotic Resistance Research | (“drinking water” OR “drinking water treatment plant” OR “water supply” OR water) AND (chlorine* OR disinfect*) AND (“bacteria resistance” OR “antimicrobial resistance” OR “antibiotic resistance” OR “ antibiotic resistant”)  <https://www.ou.edu/web/search>, <https://www.uq.edu.au>, <https://www.gu.se/en/search>, <https://amr.dundee.ac.uk/publications>, |
| Centers for Disease Control and Prevention(CDC) | ("drinking water" OR "drinking water treatment plant" OR "tap water" OR 'distribution system") AND (chlorine OR chlorination OR disinfection OR disinfectant) AND ("antimicrobial resistance" OR "antibiotic resistance" OR "antibiotic resistant")  <https://www.cdc.gov> |
| European Committee on Antimicrobial Susceptibility Testing | (“drinking water” OR “drinking water treatment plant” OR “water supply” OR water) AND (chlorine* OR disinfect*) AND (“bacteria resistance” OR “antimicrobial resistance” OR “antibiotic resistance” OR “antibiotic resistant”)  <https://www.eucast.org> |
| [Open Access Theses and Dissertations](https://oatd.org/) (OATD) | (“drinking water” OR “drinking water treatment plant” OR “tap water” OR “distribution system”) AND (“chlorine” OR “chlorination” OR “disinfection” OR “disinfectant”) AND (“antimicrobial resistance” OR “antibiotic resistance” OR “antibiotic resistant”) NOT (“sewage” OR “sludge” OR “clinical” OR “waste water” OR “wastewater”)  <https://oatd.org/oatd/search> |
| UCL DISCOVERY | Title matches "drinking water" AND Type matches any of "Article", "Proceedings paper", "Working / discussion paper", "Thesis" AND Language matches any of "English"  Title matches "antibiotic resistant" AND Type matches any of "Article", "Proceedings paper", "Working / discussion paper", "Thesis" AND Language matches any of "English"  Title matches "chlorine" AND Type matches any of "Article", "Proceedings paper", "Working / discussion paper", "Thesis" AND Language matches any of "English"  Title matches "disinfection" AND Type matches any of "Article", "Proceedings paper", "Working / discussion paper", "Thesis" AND Language matches any of "English"  <https://search2.ucl.ac.uk/s/search> |
| Food and Agriculture Organization of the United Nations (FAO) | ("drinking water" OR "drinking water treatment plant" OR "tap water" OR "distribution system") AND (chlorine OR disinfect OR disinfection OR chlorination) AND ("antimicrobial resistance" OR "antibiotic resistance" OR "antibiotic resistant")  <https://www.fao.org> |
| British Library e-theses online service | (“drinking water” OR “drinking water treatment plant” OR “water supply” OR water) AND (chlorine* OR disinfect*) AND (“bacteria resistance” OR “antimicrobial resistance” OR “antibiotic resistance” OR “antibiotic resistant”) https://ethos.bl.uk |
| The University of Oklahoma | ("drinking water" OR "drinking water treatment plant" OR "water" OR "distribution system") AND (chlorine* OR disinfect*) AND ("antimicrobial resistance" OR "antibiotic resistance" OR "antibiotic resistant")  https://www.ou.edu/web |
| Directory of Open Access Journals | ("drinking water" OR "drinking water treatment plant" OR water OR "distribution system") AND (chlorine OR disinfect OR disinfection OR chlorination) AND ("antimicrobial resistance" OR "antibiotic resistance" OR "antibiotic resistant")  https://doaj.org/search |
| Bielefeld Academic Search Engine | ("drinking water" OR "drinking water treatment plant" OR "tap water" OR "distribution system") AND (chlorine OR disinfect OR disinfection OR chlorination) AND ("antimicrobial resistance" OR "antibiotic resistance" OR "antibiotic resistant") in Title  <https://www.base-search.net> |
| University of London  British Library for Development Studies (BDLS) | ((Abstract:("Drinking Water")) OR (Abstract:("distribution system")) OR (Abstract:("tap water"))OR (Abstract:(“distribution system”))) AND ((Abstract:(chlorine)) OR (Abstract:(chlorination)) OR (Abstract:(disinfection)) OR (Abstract:(disinfectant))) AND ((Abstract:("antibiotic resistant")) OR (Abstract:("antibiotic resistance")) OR (Abstract:("resistance gene"))) NOT (Abstract:("wastewater")) NOT (Abstract:(clinical))  https://ulrls.summon.serialssolutions.com  <https://portal.research4life.org> |
